# HDAC11, an emerging therapeutic target for metabolic disorders

**DOI:** 10.3389/fendo.2022.989305

**Published:** 2022-10-20

**Authors:** Huizhen Chen, Chunguang Xie, Qiu Chen, Shougang Zhuang

**Affiliations:** ^1^ Department of Endocrinology, Hospital of Chengdu University of Traditional Chinese Medicine, Chengdu, China; ^2^ Department of Nephrology, Shanghai East Hospital, Tongji University School of Medicine, Shanghai, China; ^3^ Department of Medicine, Rhode Island Hospital and Alpert Medical School, Brown University, Providence, RI, United States

**Keywords:** HDAC11, metabolic disorders, obesity, diabetic complications, diabetes

## Abstract

Histone deacetylase 11 (HDAC11) is the only member of the class IV HDAC, and the latest member identified. It is highly expressed in brain, heart, kidney and some other organs, and located in mitochondria, cytoplasm and nuclei, depending on the tissue and cell types. Although studies in HDAC11 total knockout mice suggest its dispensable features for tissue development and life, it participates in diverse pathophysiological processes, such as DNA replication, tumor growth, immune regulation, oxidant stress injury and neurological function of cocaine. Recent studies have shown that HDAC11 is also critically involved in the pathogenesis of some metabolic diseases, including obesity, diabetes and complications of diabetes. In this review, we summarize the recent progress on the role and mechanism of HDAC11 in the regulation of metabolic disorders, with the focus on its regulation on adipogenesis, lipid metabolism, metabolic inflammation, glucose tolerance, immune responses and energy consumption. We also discuss the property and selectivity of HDAC11 inhibitors and their applications in a variety of *in vitro* and *in vivo* models of metabolic disorders. Given that pharmacological and genetic inhibition of HDAC11 exerts a beneficial effect on various metabolic disorders, HDAC11 may be a potential therapeutic target to treat chronic metabolic diseases.

## Introduction

The removal of acetyl groups from e-lysine residues in proteins (1) connected to condensed chromatin structures that inhibit gene transcription (2) is catalyzed by a class of enzymes called histone deacetylases (HDACs). Mammals currently contain 18 HDACs that are classified into two families: the Zn2+- dependent or classical HDACs, and the nicotinamide adenine dinucleotide (NAD+)-dependent HDACs or sirtuins (SIRT). According to the homology of their catalytic domains, classical HDACs are further split into three classes: class I, class II, and class IV HDACs. Class I HDACs include HDAC1, HDAC2, HDAC3, and HDAC8, whereas class II HDACs include HDAC4, HDAC5, HDAC6, HDAC8, HDAC9, and HDAC10, and class IV HDACs include HDAC11 (1).

HDAC11, the solitary member of class IV HDAC, contains an open reading frame encoding a 347-residue protein and shares sequence homology with both class I and class II HDAC proteins in the catalytic core regions. HDAC11 is highly conserved, even in invertebrates and plants as the most recently identified (3–5) and combines with other HDACs to form functional complexes (6–8). Although HDAC11 structure has still not been discovered, it has been effectively modeled from HDAC8 structure (4, 9). HDAC11 can be degraded by the proteasome system and has an unstable half-life at around four hours (10). While most class I-III HDACs are involved in deacetylating their substrates (reviewed in (11)), HDAC11 has defattyacylase activity in addition to its deacetylase activity. In fact, as the only HDAC member that has a clear predilection for the removal of long-acyl instead of acetyl groups (12, 13), HDAC11 is the family’s most effective fatty deacetylase (9). It has been reported that the efficiency of HDAC11 defattyacylase activity is greater than 10,000 times its deacetylase activity (13). The activation of HDAC11 can be triggered by physiologic levels of free fatty acids and their metabolites (9).

HDAC11 is expressed in multiple organs and distributed in diverse organelles. It is primarily expressed in heart, kidney, smooth muscle (3), skeletal muscle (14–16), brain (3, 15, 17–20), testis (14, 21) and gall bladder (22). At cellular level, HDAC11 is abundant in the mitochondria of skeletal muscle cells, brain synapses (15), and the centrosomes of neurons from the dentate gyrus (19). But it locates predominantly in the cytoplasm of embryonic astrocyte precursors and mature cells (23), and the nucleus of activated astrocytes and oligodendrocytes (24). HDAC11 can be found both in the cytoplasm and the nucleus of newly isolated and unstimulated Treg cells (24), immature astrocytes and oligodendrocytes (23), retinal ganglion cells (25) and preadipocytes (26). In addition, HDAC11 is highly expressed in the rat brain, and pancreatic β cells (27).

Emerging evidence has indicated that HDAC11 is critically involved in physiological and pathological processes. HDAC11 has a variety of physiological functions, including immunomodulation (24, 28–36), genomic stability (21, 37–39), cell cycle progression (21, 40, 41), and nervous system development (42). Pathologically, HDAC11 plays a role in epithelial barrier dysfunction (43–45) and ischemic injury (46–48) and requried for the growth of several tumors (49–56), such as hepatic carcinoma (57–61), and lung cancer (62, 63). Moreover, it contributes to the development of some other diseases (56, 64), including hepatitis B (65–67) and age-related macular degeneration (68).

In the past two decades, HDACs have been revealed to be implicated in the regulation of multiple metabolic processes and pathogenesis of some metabolic disorders. For example, most class I HDAC members are associated with insulin resistance, energy metabolism and glucose homeostasis, and contribute to the pathogenesis of diabetes and its associated complications (69, 70), and obesity (71). Class II HDACs are required for regulating the transcription of genes associated with glucose homeostasis and hepatic gluconeogenesis (72). Moreover, HDACs are involved in the regulation of several events related to the pathogenesis of diabetes (i.e. oxidative stress, inflammation and fibrosis) and its associated complications (70, 73). Very recently, HDAC11 has been linked to the pathogenesis of obesity (74), diabetes, and diabetic complications (64, 75). Given that global deletion of HDAC11 in mice does not affect their development and health (24), pharmacological inhibition of HDAC11 could be a potential therapeutic approach for the treatment of metabolic disorders. In this review article, we summarize the role and possible mechanisms of HDAC11 in metabolic disorders, including obesity, metabolic inflammation, and diabetes and its complications, and provide detailed information about HDAC11 inhibitors developed so far.

## HDAC11 in obesity

Obesity is an excessive fat gain due to unbalanced energy intake and consumption (76), and its prevalence rises yearly in children and adults (77). HDAC11 is related to obesity in multiple ways.

HDAC11 participates in the regulation of adipogenesis. The differentiation of adipocytes is strictly controlled. Mature adipocytes are differentiated from mesenchymal precursor cells. Several essential adipose transcription factors, such as peroxisome proliferator‐activated receptor γ (PPARγ), CCAAT‐enhancer-binding protein β, and sterol regulatory element‐binding proteins regulate this process (78–80). It has also been reported that various HDACs, in particular, HDAC11 are critically involved in adipogenic differentiation (81–83). Silencing the HDAC11 gene by small interfering RNA results in reduced perilipin, adipoq, and PPARγ2 expression, and decreased formation of intracellular lipid droplets (84). By the use of HDAC11-KO mice and adipocytes from WT and HDAC11 KO mice exposed to FT895, it was also found that HDAC11 binds to a nearby gravin-α region and demyristoylates those spots. Gravin-α lysine myristoylation in brown and white adipocytes is necessary for the signal through β2- and β3-adrenergic receptors (β-ARs). Gravin-α lysine myristoylation induces the expression of protective thermogenic genes by directing β-ARs to lipid raft membrane microdomains and stimulating activation of PKA and its downstream signaling. These results establish reversible lysine myristoylation as a pattern of GPCR signaling regulation and emphasize the importance of HDAC11in regulating adipocyte phenotypes (85).

HDAC11 is essential for regulating the balance of brown adipose tissue (BAT) and white adipose tissue (WAT) (86). The WAT is the body’s greatest energy storage tissue, and can secretes cytokines and adipokines as part of its endocrine function; BAT is imperative in maintaining body temperature in newborns’ nonshivering thermogenesis (87–90). A role for HDAC11 in regulating adipose tissue and thermogenic capability has been suggested by the fact that HDAC11 is more expressed in WAT than BAT and that deletion of HDAC11 in mice enhances the development of BAT and “browning” of WAT (26). These are essential changes as WAT contributes to obesity by storing extra energy as fat in the body, while BAT is capable of turning fat into energy (90). Meanwhile, In HDAC11‐knockout (KO) mice, the histological study of BAT reveals a compacted tissue size with noticeably smaller lipid droplets (75). Mouse hepatic cell line AML12 with HDAC11 knockdown exhibits enhanced metabolic activity and oxygen consumption due to improved lipid oxidation capability (75). which is consistent with previous observations in skeletal muscle tissue (14).

Mechanistically, uncoupling protein 1 (UCP1), a mitochondrial long-chain fatty acid/H+ symporter, and PGC1-α, a primary regulator of mitochondrial biogenesis, are both downregulated by HDAC11 to inhibit the BAT transcriptional program (26). HDAC11 deletion increases metabolic pool clearance, thermogenic capability, UCP1 expression in BAT, and energy expenditure. Through its physical interaction with BRD2(an enhancer regulating Ucp1 gene) (26), HDAC11 inhibits the thermogenic gene program. HDAC11 inhibition increases oxygen consumption and boosts adiponectin, a hormone that controls fatty acid oxidation, blood glucose levels, and stimulates lipid metabolism by activating the adiponectin-AdipoR-AMPK pathway (75).

Recent studies have also shown that HDAC11 is a critical regulator of the body’s overall metabolism. HDAC11 KO mice exhibit higher body temperatures than wild type (WT) controls both at room temperature (22°C) and during a 24-hour cold challenge (4°C), which is correlated with higher metabolic rate and oxygen consumption (26, 75). Importantly, HDAC11-deficient mice show alleviated hypercholesterolemia, hepatic steatosis and liver damage (26, 75).

Altogether, these results suggests that HDAC11 is a new metabolic regulator, lowering its levels might improve cells’ ability to adapt to an elevated energy requirement under stressful circumstances. Furthermore, as a result of the considerable rise in metabolic rate and oxygen consumption caused by HDAC11 inhibition, there is an increase in lipid oxidation and energy expenditure. Therefore, HDAC11 would be a prospective therapeutic target for obesity and the related metabolic effects.

## HDAC11 in diabetes

### Diabetes

Diabetes is a metabolic, chronic, multisystem disease and chronic exposure to hyperglycemia eventually leads to multiple complications, such as diabetic nephropathy, cardiovascular disease, retinopathy and neuropathy with considerable impact on the quality of life and overall life expectancy.

HDAC11 is essential for preserving insulin sensitivity and glucose homeostasis. In mice fed with high fat diet (HFD), HDAC11 deletion significantly decreases blood insulin levels, stabilizes blood glucose, and greatly reduces blood glucose levels after insulin challenge, thereby enhancing glucose tolerance and ameliorating diabetes (75). In addition, adiponectin significantly increases in HDAC11 KO mice (91). By uisng adiponectin-knockout mice fed on a HFD or either regular chow, it has been demonstrated that adipoR2-peroxisome proliferator-activated receptor α (PPARα) and adipoR1-AMP-activated protein kinase (AMPK) pathways play a major role in adiponectin signaling in the liver (91). The vital energy sensor AMPK has been linked to the control of the hepatic metabolic processes, such as gluconeogenesis. Increased energy expenditure, improved glucose tolerance, and lower plasma cholesterol levels all result from AdipoR2 KO. Lysophospholipids are one of adiponectin’s targets, and they are upregulated by a high-fat diet (HFD) and tend to cause hypertriglyceridemia, decreased glucose tolerance, and insulin resistance (91).

### Diabetic nephropathy

Diabetic nephropathy (DN) is a serious complication of diabetes. It presents as localized kidney inflammation and fibrosis that lead to structural remodeling (92–94).

Although there is no report about the role of HDAC11 in DN so far, HDAC11 is vital in the response to renal inflammation and fibrosis. Plasminogen agonist inhibitor type 1, a physiological inhibitor of fibrinolysis (PAI‐1), is evelvated in DN (95). Excess PAI‐1 lead to the accumulation of extracellular matrix proteins, whereas PAI‐1 deficiency protected the kidney from injury-induced fibrosis (96). In a murine model of renal ischemia/reperfusion (I/R), increased testosterone can decrease the ability of HDAC11 to bind to PAI-1 promoter, leading to increased histone 3 acetylation and PAI-1 expression and accelerated I/R-induced renal injury (46, 47). Moreover, HDAC11 expression are increased in the kidneys in animal models of renal fibrosis induced by unilateral ureteral obstruction and angiotensin II by suppressing Kruppel-like factor 15, an anti-fibrogenic factor (97). Since renal inflammation and fibrosis contribute to the pathogenesis of DN, it is speculated that HDAC11 would also play a role in the development of DN. Further studies are needed to address this issue.

### Diabetic cardiopathy

Type 2 diabetes and cardiovascular diseases are predisposed to by obesity (98). Increased body weight can, in fact, cause metabolic changes in cardiomyocytes that switch them from processing fatty acids to sugar, which adds to lipid storage in the pericardium and, as a severe consequence of type 2 diabetes, causes myocardial infarction (99). Interestingly, inhibition of HDAC11 activity could prevent or ameliorate diabetic cardiomyopathy. In apoE mice fed with HFD, atherosclerosis and blood lipid levels have recently been shown to be alleviated by HDAC11-AS1. HDAC11-AS1 improves lipoprotein lipase (LPL), a crucial rate-limiting enzyme involved in triglyceride (TG) hydrolysis, *via* controlling adropin histone deacetylation both *in vitro* and *in vivo (* 100). Another study shows that suppression of HDAC11 enhances the prevention of pyroptosis in human umbilical vein endothelial cells (HUVECs) triggered by TNF-α, indicating that vascular endothelial pyroptosis might be prevented through downregulation of HDAC11 related signaling pathways in atherosclerosis (AS) (101). In addition, a fructose injury-induced mouse model of diabetic heart failure that lacks HDAC11 had lower levels of apoptosis, dyslipidemia, inflammation, and oxidative stress (102). HDAC11 has also been suggested to be an essential regulator in heart failure (103). Therefore, HDAC11 contributes to the pathogenesis of diabetic Cardiopathy.

## HDAC11 in metabolic inflammation

Metabolic disorders are closely associated with chronic mild inflammation (104–106). Most obese people develop inflammation in their adipose tissue, like chronically damaged tissue, along with immune cell remodeling and infiltration. During the early phases of adipose swelling and the progression of chronic obesity, inflammation is induced, and the immune system is irreversibly changed into a proinflammatory phenotype (107). Changes in adipose tissue function are related to obesity, and the loss of adipocytes also contributes to chronic mild inflammation (104). The regulating function of HDAC11 in metabolic inflammation is crucial.

HDAC11 regulates metabolic inflammation primarily through the control of the IL-10 released by antigen-presenting cells (APCs) (28). Inhibition of HDAC11 causes macrophages to express more IL-10, whereas overexpression of HDAC11 reduces IL-10 expression (108, 109). In addtion, HDAC11 overexpression in APCs is efficient in reactivating tolerant T cell responses and CD4+ T cells specific for antigens. And APC had the reverse result when HDAC11 expression was absent (33). Conversely, suppression of HDAC11 resulted in impaired antigen-specific expression, increased IL-10 expression, downregulated IL-12 expression and immune cell expression (such as myeloid-derived suppressor cells, neutrophils, and T cells), leading to immune tolerance (110, 111). In addition, muted HDAC11 transcripts boosted the synethesis of IL-17 and TNF in the supernatants of HL cells (112). Moreover, liver immune tolerance is regulated by HDAC11 through TNF‐α, interferon‐γ, IL‐2, and IL‐4 (80, 90, 113–120).

## HDAC11 inhibitors

Most HDACi are pan-HDACi that target multiple HDACs with different nanomolar potency. Zinc-dependent catalytic processes are shared by Classes I, II, and IV HDACs. Many pan-HDACi have been synthesized, including Aes-135 (121), AR-42, belinostat (PXD101,PX105684), fimepinostat (CUDC-907) (9), FT895 (122), M344(D237, MS344), Panobinostat (LBH589, NVP-LBH589), pracinostat (SB939), dacinostat, quisinostat (JNJ-26481585), trichostatin A (TSA), vorinostat (34) (SAHA, MK0683), mocetinostat (123)(MGCD0103), tucidinostat (Chimade, HBI-8000, CS055), trapoxin A (124)(TpxA, C34H42N4O6), garcinol (125), romidepsin (126). Recently, Compound 8, a newly designed novel HDAC6 selective inhibitor with 2-mercaptoquinazolinone as the Cap Moiety, has displayed stronger inhibition activity against HDAC11 than Belinostat (127). The toxicity caused by general inhibition of HDACs restricts their potential utility. Among the pan-HDAC inhibitors, garcinol shows more HDAC11 selectivity and efficiency than other HDACs (125). The deacetylase and demyristoylase activities of HDAC11 are also suggested to be effectively inhibited by Fimepinostat (9). At concentrations of 0.02, 0.2, and 2μM, respectively, Suberoylanilide hydroxamic acid (SAHA) could suppress 10, 50, and 90% of HDAC11 activity (34). Additionally, it has been noted that trichostatin A (TSA) and romidepsin have a nanomolar potency toward HDAC11 (126). However, pracinostat, dacinostat, mocetinostat, quisinostat, trapoxin A, and trichostatin A have been found not as efficient in inhibiting HDAC11 deacetylation activity as reported before (9). Unexpectedly, butyrate, valproate, SAHA, and TSA could trigger myeloid cells to express HDAC11 (128). And low doses of MS275 have been found to show agonistic actions (129).

Elevenostat (JB3-22) (21, 24), SIS7, SIS17 (130), and FT895 (122) are selective HDAC11 inhibitors. Nevertheless, the inhibitory capacity of elevenostat (JB3-22) on myristoylated and acetylated peptidic derivatives is extremely poor (9). To date, the HDAC11 inhibitors that are considered to be the most potent and selective are SIS17 and FT895. SIS17 is better than FT895 and SIS7 in terms of its cell permeability and metabolic stability (60), while FT895, SIS17, SIS7 can all inhibit HDAC11’s demyristoylase activity (130).

Though several HDAC11 inhibitors have been developed, only FT895 (85, 122), romidepsin (131), and quisinostat (97, 131, 132) have been reported to be utilized in animal studies. To explore the pharmacokinetic properties of FT895, it was injected to male Balb/c nude mice *via* i.v. at 1mg/kg) or i.p. at 5 mg/kg. After i.v. dosing, with a t1/2 of 9.4 hours, FT895 exhibits a high volume of distribution and a moderate clearance (42 mL/min/kg). In comparison, FT895 dosed intravenously has enhanced exposure, a similar half-life (10.2 h), a bioavailability of 81%, and sustains free drug levels above the cellular half-maximal inhibitory concentration (IC50) for up to 4 hours (122). Quisinostat (10 mg/kg Monday, Wednesday, and Friday) and romidepsin (0.3 mg/kg, 1 mg/kg, or 3 mg/kg Monday, Friday) were administered intraperitoneally (i.p.) for one week to tumor-bearing athymic NOD.Cg-Prkdcscid Il2rgtm1Wjl/SzJ (NSG) mice. Romidepsin has unacceptable toxicity at 3 mg/kg; anemia and aspartate aminotransferase elevations are a result of 1 mg/kg dosing; Without causing considerable weight loss (>20%) or neurotoxicity, both 0.3 mg/kg and 1 mg/kg are tolerated. Treatment with quisinostat (10 mg/kg Monday, Wednesday, Friday) shows no systemic toxicity (131). Similarly, in BALB/c nude mice and NOD/SCID mice, quisinostat (3 and 10 mg/kg/day, i.p) has been used to treat tongue and esophageal squamous cell carcinoma (132, 133) and malaria (134). Romidepsin is also used in C57BL/6 (0.03mg/kg twice a week) (135, 136) and BALB/C(1mg/kg/2days) (137) mice for cancer treatment. Therefore, FT895, quisinostat and romidepsin are tolerable and safe *in vivo*.

In addition, quisinostat and romidepsin have been tested in clinical trials. The maximum tolerable dose of quisinostat for the treatment of cancer in patients is 10 mg administered orally three times per week along with bortezomib and dexamethasone, with median progression-free survival (PFS) 8.2 months and median duration of response 9.4 months (138). Combined with 5-azacytidine (AZA), romidepsin (14 mg/m^2^, day 8,15,22, per 35 days, IV) is used to treat peripheral T-cell lymphomas (PTCL) with the overall survival not met (at a median follow-up of 13.5 months), and the median progression-free survival (PFS) 8.0 months, duration of response 20.3 months (139). Romidepsin has also been reported to treat HIV-1-infected patients with a 5 mg/m^2^ dosage as a 4 hour infusion (140).

Thus, taking effectiveness, selectivity, toxicity, half-life, tolerance and survivability *in vivo* into consideration, FT895 exhibits pharmacokinetic properties that are reasonable *in vivo* research, and the most significant potential to advance into clinical trials.

## Conclusion and perspectives

**Figure 1:**
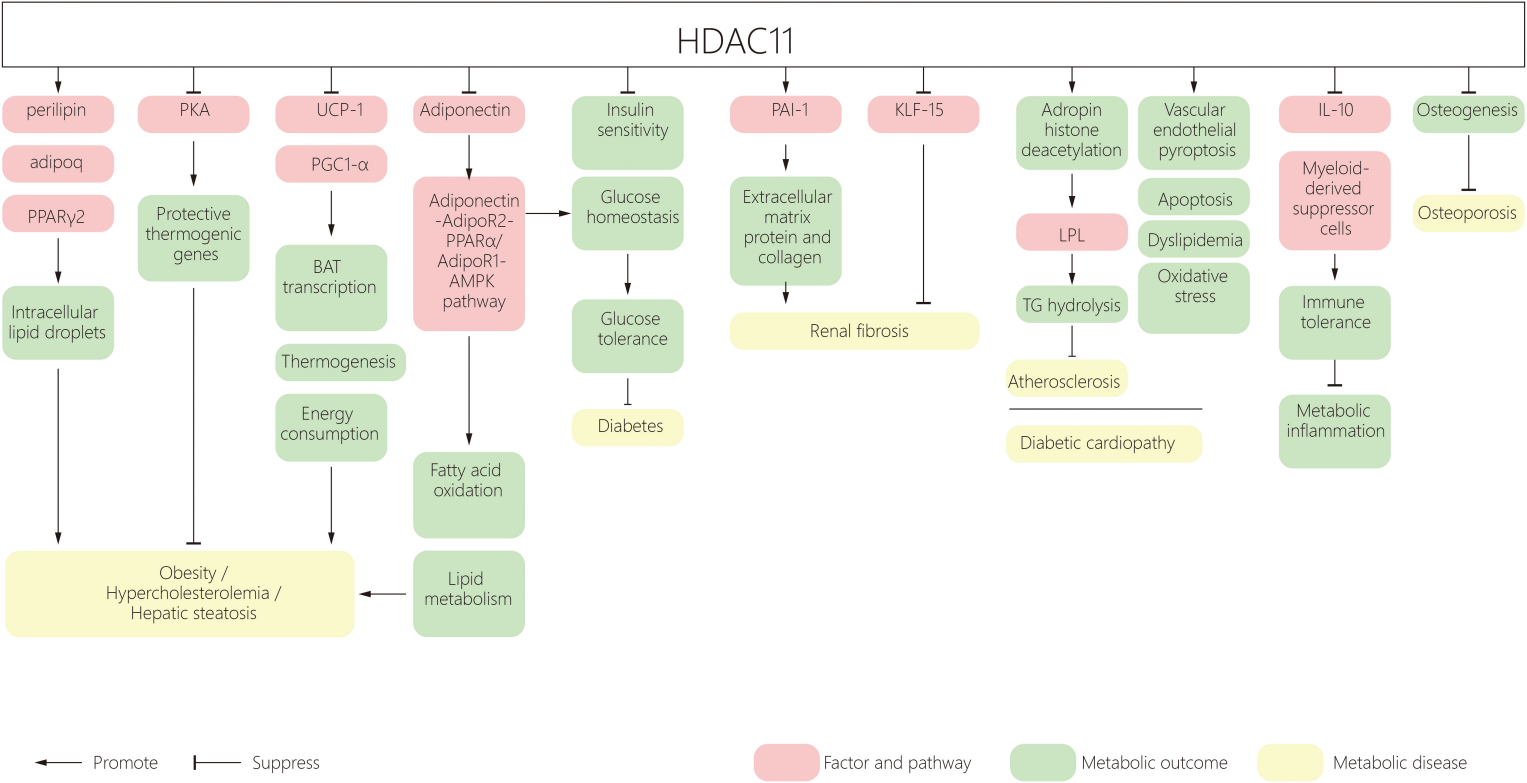
** **A schematic diagram of HDAC11 and its effect on metabolic disorders. HDAC11, Histone deacetylase 11; PPAR, peroxisome proliferator‐activated receptor; BAT, brown adipose tissue; UCP1, uncoupling protein 1; AMPK, AMP-activated protein kinase; PAI‐1, Plasminogen agonist inhibitor type 1; AP-2α, activator protein 2α; KLF15, Kruppel-like factor 15; LPL, Lipoprotein lipase; TG, triglyceride.

The incidence of metabolic disorders is increasing worldwide, ranging from obesity to type 2 diabetes, leading to complications in the heart, kidney, retina, bone, skin and foot. HDAC11 participates in many aspects of metabolic diseases. HDAC11 mediates obesity and metabolic syndrome by regulating adipogenesis, increasing energy consumption and promoting lipid metabolism. It also contributes to adipose tissue inflammation by regulating immune responses and insulin resistance. HDAC11 was shown to have inhibitory roles in the development of diabetic cardiovascular disease. (**Figure 1**) In addition, a recent study shows that HDAC11 contributes to osteoporosis susceptibility and reduced peak bone mass through a mechanism of 11β-HSD2’s low-functional programming. This is triggered by corticosterone through GR/HDAC11 signaling, which amplifies the effect of corticosterone on inhibiting the function of BMSCs in osteogenesis (141, 142). However, studies on diabetic osteoporosis, lipoid nephrosis, fatty liver disease and obesity cardiomyopathy are still lacking. As such, further research on the effect of HDAC11 on metabolic diseases is required.

Studies listed in **Table 1** have shown the well-tolerated HDAC11 global deletion in mice, suggesting that its inhibition or depletion is without apparent side effects. Currently, toxicity and safe doses of HDAC11 inhibitors are far from clear and none of the inhibitors have been used in patients with metabolic disorders. Thus, more studies on the safe dosage and toxicity of HDAC11 inhibitors in animal models are needed before advancing them to human clinical trials. In addition, development of more effective HDAC11 inhibitors with enhanced selectivity is worth investigating.

**Table 1:** The role of HDAC11 in metabolic disorders.

Related metabolic diseases	Animal model	Approach (HDAC11 inhibitor/KO)	Main findings	Reference
Obesity	/	KO	HDAC11 deficiency improves muscle function, fatigue resistance and muscle strength with enhanced mitochondrial content and oxidative myofibers by lowering acylcarnitine levels, activating the AMP-activated protein kinase-acetyl-CoA carboxylase pathway and stimulating a glycolytic-to-oxidative muscle fiber switch.	(14)
	Cold challenge HFD	KO	HDAC11 deficiency increases BAT abundance and function, metabolism, and glucose tolerance resultant from acute high fat feeding.	(26)
	HFD	KO	HDAC11 deficiency enhances glucose tolerance and insulin sensitivity, attenuates liver damage, hepatosteatosis and hypercholesterolemia by boosting energy expenditure through promoting thermogenic capacity.	(75)
	/	KO	HDAC11 demyristoylates gravin-α in adipocytes, leading to protective thermogenic gene expression.	(85)
Diabetic nephropathy	Renal fibrosis	Inhibitor (quisinostat)	Inhibition of HDAC11 attenuates renal fibrosis, blocks the pro-fibrogenic response induced by Ang II through interaction with activator protein 2 to activate KLF15 transcription.	(97)
Diabetic/obesity related cardiopathy	Atherosclerosis	Inhibitor (HDAC11) antisense	HDAC11-AS1 reduces blood lipid levels and atherosclerosis of apoE-/- mice fed with HFD by enhancing LPL and TG metabolism	(100)
	Cardiac dyslipidemia	KO	HDAC11 depletion elevates blood pressure, reduces the inguinal fat-pad mass and body weight, with improved cardiac function, dyslipidemia, enhanced SOD activity.	(102)
Metabolic inflammation	Rat orthotopic liver transplantation	Inhibitor (HADC11-shRNA)	HDAC11 inhibition promotes the expression of IL-4 and IL-10, reduces IFN-γ, TNF-α, and IL-2 levels, and induces tolerance.	(113)

HDAC11, histone deacetylase 11; WAT, white adipose tissue; BAT, brown adipose tissue; UCP1, uncoupling protein 1; KO, knockout; HFD, high fat diet; KLF15, Kruppel-like factor 15.

## Author contributions

HC drafted the article. The manuscript was edited by SZ, CX, and QC. After reviewing the manuscript, all authors gave their approval for publication.

## Funding

This study is supported by US National Institutes of Health (2R01DK08506505A1 to SZ), the National Natural Science Foundation of China grants (81670623 and 81830021 to SZ), and National key R&D Program of China (2018YFA0108802 to SZ).

## Acknowledgments

We are grateful to Dr. George Bayliss from Brown University for editing this manuscript.

## Conflict of interest

The authors declare that the research was conducted in the absence of any commercial or financial relationships that could be construed as a potential conflict of interest.

## Publisher’s note

All claims expressed in this article are solely those of the authors and do not necessarily represent those of their affiliated organizations, or those of the publisher, the editors and the reviewers. Any product that may be evaluated in this article, or claim that may be made by its manufacturer, is not guaranteed or endorsed by the publisher.

## Abbreviations

HDAC11, Histone deacetylase 11; HDACs, Histone deacetylases; NAD+, nicotinamide adenine dinucleotide; SIRT, sirtuins; AMD, age-related macular degeneration; MS, multiple sclerosis; PPAR, peroxisome proliferator‐activated receptor; β-ARs, β-adrenergic receptors; WAT, white adipose tissue; BAT, brown adipose tissue; UCP1, uncoupling protein 1; WT, wild type; KO, knockout; AMPK, AMP-activated protein kinase; HFD, high fat diet; DN, Diabetic nephropathy; PAI‐1, Plasminogen agonist inhibitor type 1; AP-2α, activator protein 2α; KLF15, Kruppel-like factor 15; LPL, Lipoprotein lipase; TG, triglyceride; HUVECs, human umbilical vein endothelial cells; AS, atherosclerosis; APCs, antigen‐presenting cells; TSA, trichostatin A; TpxA, trapoxin A; SAHA, Suberoylanilide hydroxamic acid; i.p., intraperitoneal; IC50, half maximal inhibitory concentration; PFS, progression-free survival; AZA, 5-azacytidine; PTCL, Peripheral T-cell lymphomas; PFS, progression-free survival.
